# Supplementary material for: A large-scale view of marine heatwaves revealed by archetype analysis
Source: Nat Commun. 2022 Dec 21;13:7843. doi: 10.1038/s41467-022-35493-x (PMC9772210; doi:10.1038/s41467-022-35493-x)
Supplement: Supplementary file 1 — Supplementary Information [file 41467_2022_35493_MOESM1_ESM.pdf]

Supplementary Information for A Large-Scale View of Marine Heatwaves Revealed by Archetype Analysis

Christopher C. Chapman[1,2], Didier P. Monselesan[1], James Risbey[1], Bernadette M. Sloyan[1,2], and Ming Feng[2,3]

[1] *CSIRO Oceans and Atmosphere, Hobart Marine Laboratories, Castray Esplanade, Hobart, Tasmania, Australia*
[2] *Center for Southern Hemisphere Ocean Research, Hobart Marine Laboratories, Castray Esplanade, Hobart, Tasmania, Australia*
[3] *CSIRO Oceans and Atmosphere, Indian Ocean Marine Research Center, Crawley, Western Australia*

December 6, 2022

Supplementary Case Studies

In this section, we provide further case studies to illustrate the utility, and limits of the 'outside-in' approach based on archetype analysis (AA). While the main text focuses on marine heatwaves, here we present two case studies concerning the cold extremes, known as marine coldspell, and a case study showing how AA is unable to reflect regional marine extremes likely induced by small scale local processes and not broad-scale processes such as El-Niño or La-Niña.

Marine Cold Spells in the Southeast Indian Ocean

In the main text, we discuss marine heatwaves in the southeast Indian ocean off the coast of Western Australia. This region is often discussed in relation to marine heatwaves due to the high profile of the devastating 2011 event that reached the 'extreme' category[1]. However, recent attention has turned to marine coldspells in the region, as these events can also have important effects on fisheries and ecological communities[2]. These analyses have shown that, much like marine heatwaves in the region, marine coldspells tend to *cluster*, with multiple events occurring during a relatively brief period of time.

In Supplementary Fig. 1, we show the composite average of marine coldspells for a representative point in the south-east Indian ocean (Supplementary Fig. 1**a**), together with the peak day of two events, corresponding to the a

'strong' event in June 2017 (Supplementary Figs. 1**b**) and a 'moderate' event in June 2018 (Supplementary Figs. 1**d**), together with the two best-matching archetypes (Supplementary Figs. 1**c,e**), both of which show a broad similarity the daily snapshots and the composite average.

The SST anomaly at the representative location, shown in Supplementary Fig. 1**f**, shows clear low-frequency variability, with periods of below average SST persisting for months or years at a time. Examples of these periods are 1986-1988, 1990-1992, 1997, 2003-2004 and 2017-2019. Unsurprisingly, these periods correspond to periods relatively frequent marine coldspells. The affiliation time-series of the two best matching archetypes, showing in Supplementary Fig. 1**g**, appears to capture this low frequency variability, particularly archetype #1 (archetype # 2 appears to correspond more commonly to isolated marine coldspell periods). For example, the multi-year marine cold-spell described by Feng et al. 2021[2], that occurred over the period 2016 to 2020, can be seen to correspond to a period where the affiliation of both best-matching archetypes is consistently higher than 0.4.

Zooming out to investigate the teleconnection patterns, shown in Supplementary Fig. 2. We note that, in contrast to the marine heatwave case-study presented in the main text, there is no strong SST signature in the tropical Pacific. In fact, the affiliation time-series is positively correlated with the multivariate ENSO index at a peak lag of +10 months (Supplementary Figs. 2**d,e**), suggestive that widespread cold temperatures in the southeast Indian ocean occur 8-12 months prior to the onset of El-Niño. The anomalous atmospheric circulation, shown in Supplementary Fig. 2**b**, indicates widespread anomalously equatorward winds over the region of interest, and likely contributes to the cooler than average temperatures. However, correlation with the Southern Annular Mode (SAM) index is weak and the spatial patterns of mid-tropospheric geopotential height anomalies are not strongly reminiscent of the canonical SAM.

Tasman Sea and East Australian Current Marine Heatwaves

In the main text, we have employed case-studies to demonstrate the utility of archetypal analysis in identifying large-scale 'extreme' modes of variability that can influence local environmental conditions sufficiently to induce (or at least contribute) to the occurrence of marine temperature extremes. However, the conditions at any particular location are obviously influenced by both broad-scale (or remote) and local factors. In the following case-study, we illustrate two cases where local dynamics, not broad scale dynamics, are the dominant influence.

We take the well studied 2016 Tasman Sea marine heatwave as our first example. This event occurred in the southern Tasman Sea, centered to the east of Tasmania, starting in early September 2015 and lasting for approximately

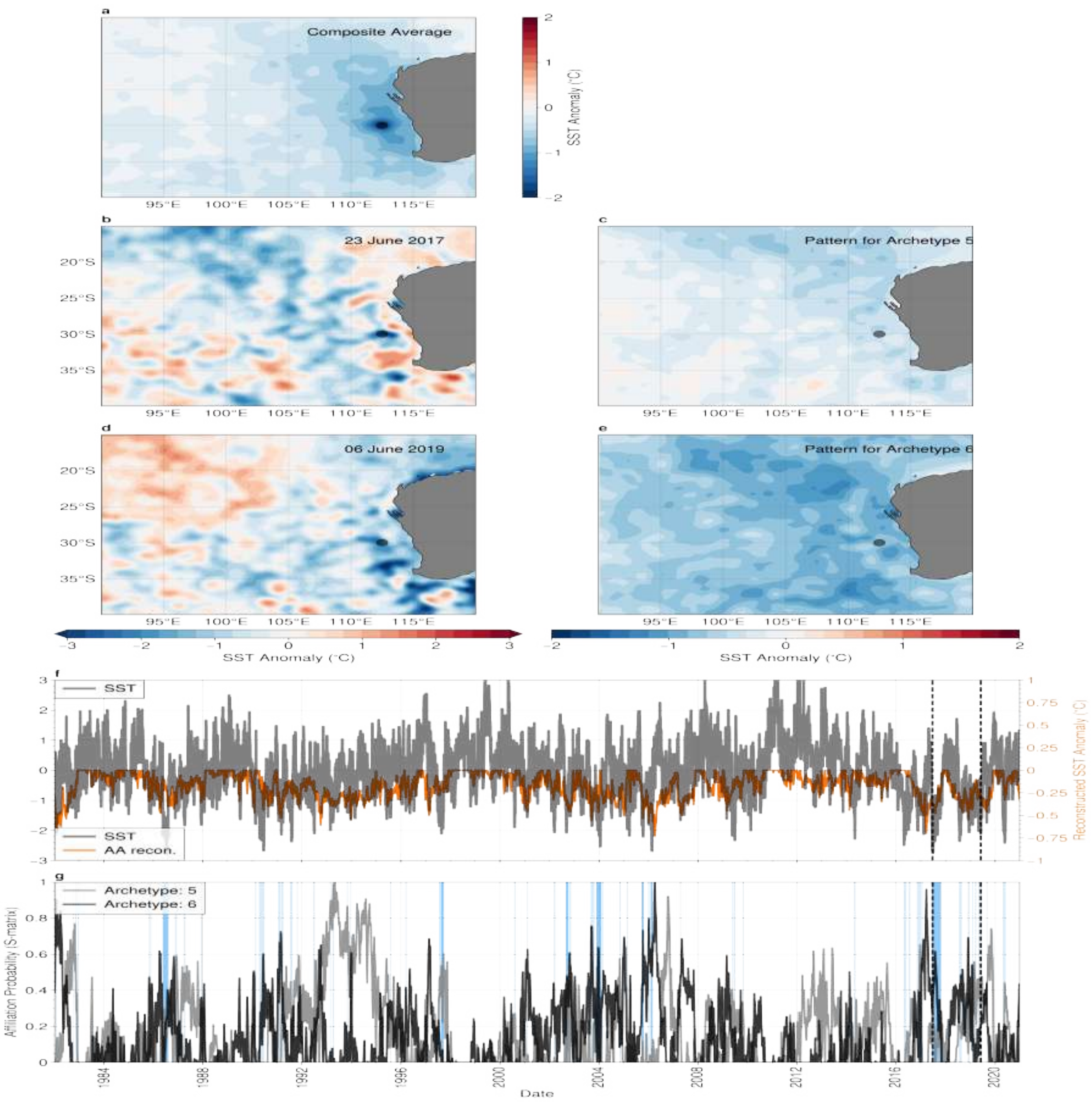

Supplementary Figure 1: **The relationship between Marine Coldspells and Archetypes #5 and #6 in the southeast Indian ocean** : **a** Sea-surface temperature (SST) anomaly composite average of all marine coldspells at a representative location (at 30°S,112.5°E, shown as a grey dot) in the south-east Indian Ocean; **b,d** snapshot of SST anomalies for the peak for the peak day of the strong marine coldspell event on the 23rd of June, 2017, and the moderate event on the 6th of June 2018; **c,e** the SST anomalies for best matching archetypal pattern (archetype #5 archetype #6); **f** time-series of SST anomalies (black) and the reconstruction from archetype 3 (orange) at the representative location shown in panels **a**–**c** ; **e** time-series of archetype affiliation probability for archetype 3. Colored bands in panels **d,e** indicate marine coldspell occurrences, coded by the severity category described in *Hobday et al. 2018*[3].

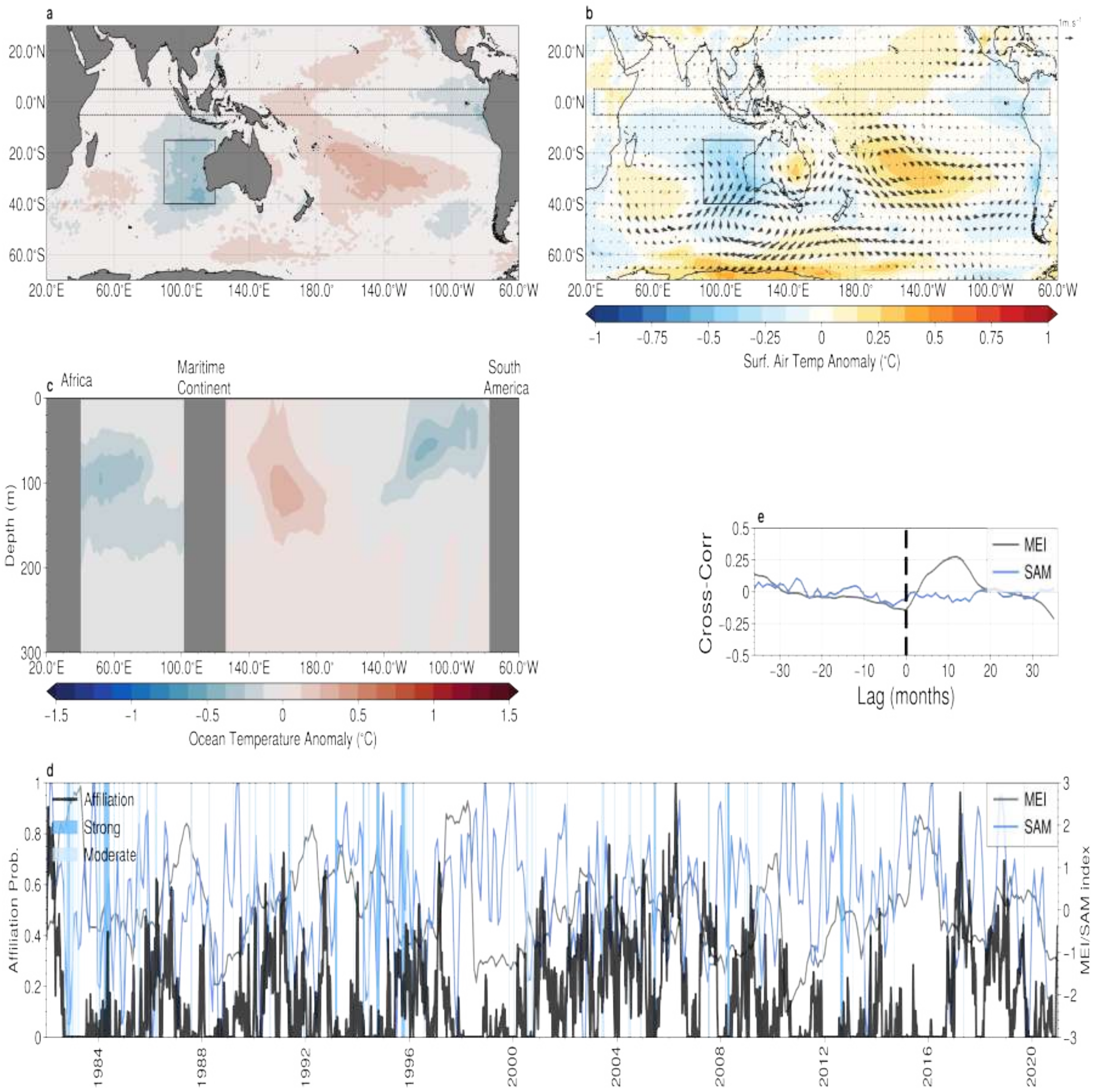

Supplementary Figure 2: **Teleconnections Associated with Marine Cold-spells in the Southeastern Indian Ocean**: **a** Sea-surface temperature (SST) anomaly; **b** surface air temperature (colours) with anomalous mid-tropospheric (500hPa) geopotential height (contour lines) and winds (vectors); and **c** equatorial subsurface temperatures, associated with archetype #3, the best-matching archetype for marine heatwaves in the southeast Indian Ocean.**d** The affiliation time-series (solid black) together with the multivariate El-Niño index (MEI, grey) and the Marshall Southern Annular Mode (SAM) index (blue). Periods of marine heatwaves are indicated by red shading. **e** the lagged cross-correlation between the affiliation time-series and the MEI (gray) and the Marshall SAM index (blue). Negative lags correspond to the MEI/SAM index leading the affiliation.

251 days to finish in May 2016[4]. Supplementary Fig. 3**a** shows the peak day of this event (8th February 2016) at a representative location (42.75°S,148.5°E, shown as a grey circle in Figs 3) along with the composite average of all events at that location (Supplementary Fig. 3**b**). Both the peak day snapshot and the composite average have similar spatial structures, which suggests that most marine heatwaves in the region have a similar spatial structure.

In contrast, the spatial structure of the best matching archetype (Supplementary Fig. 3**c**) does not show the same spatial structure as either the daily snapshot or the composite average of marine heatwaves near Tasmania. While the time-series of the SST anomaly (Supplementary Fig. 3**d**) and the affiliation time-series of the best-matching archetype show correlation in later part of the time-period (from 2008 onward) with a particularly notable co-occurrence of SSTs and an affiliation probability near 1 during the 2015-2016 marine heat wave event. However, the spatial patterns associated with the best-matching archetype show little similarity to those associated with southern Tasman sea marine heatwaves. Instead of localised warm SSTs around Tasmania, the archetype shows a broad region of elevated SSTs in the Tasman and Coral Seas, extending from the east coast of the Australian continent to around 180° longitude. Prior to 2008, periods with high affiliation probabilities are not consistently associated with marine heatwaves at the representative location. For example, the affiliation probability reaches 1 in 1998, a time period associated with *lower* than average SSTs.

In a similar vain, we plot the same marine heatwave metric for events in the East Australian Current (EAC) (Supplementary Fig. 4), at 30°S, near the city of Coffs Harbour. Marine heatwaves in this region have been attributed to a combination of local oceanographic factors, such as eddy interaction with the EAC, and atmospheric effects[5]. In this case, the spatial structure of SSTs for the peak of the most intense event (4th February 2017, Supplementary Fig. 4**a**), the composite average of all events at this location (Supplementary Fig. 4**b**) and the best-match archetype (Fig. 4**c**, as in the southern Tasman Sea case study above) are similar, with a broad pattern of high SST anomalies over the Tasman Sea region, from the eastern Australian coastline to a longitude of $\sim$180°, although we note that the strongest SSTs are found along the Australian coastline in both the single day snapshot and the composite average of all events, which is not apparent in the SST pattern associated with the archetype. However, investigating the time-series (Supplementary Fig. 4**d,e**) shows limited correlation between the affiliation time-series and the SST time-series at the representative location, with the exception of the period around 1998 and, to a much lesser extent, around 2010, which showed both an elevated affiliation probabilities and anomalously high SSTs. Marine heatwave periods tend to cluster in the later half of the time-series, consistent with the enhanced SSTs in the region as a result of continued global warming[6].

The teleconnections associated with best-matching archetype are shown in Supplementary Fig. 5. The SST pattern in the equatorial Pacific, together with the peaks in the affiliation time-series in the years 1998 and 2016, strongly

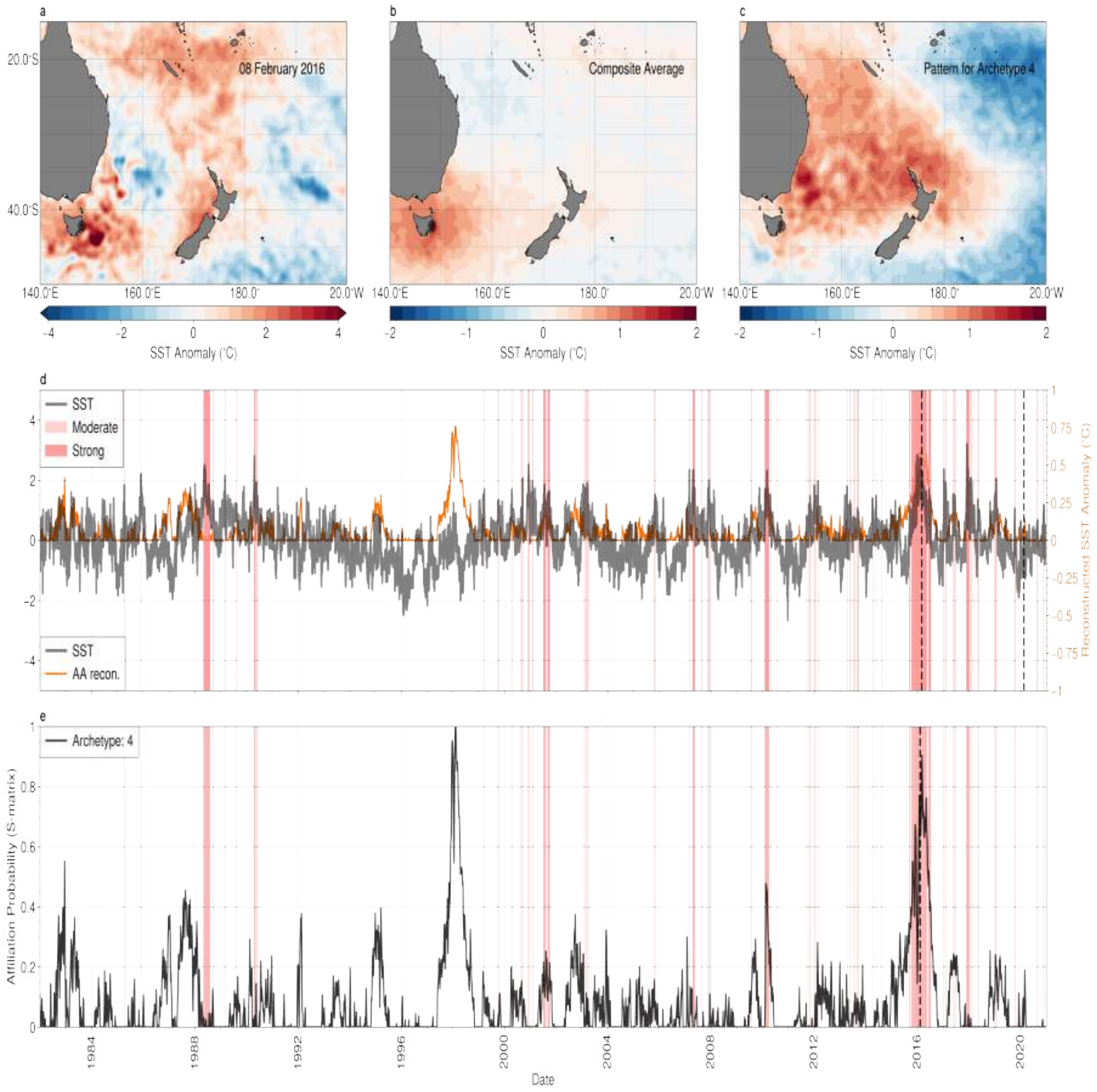

Supplementary Figure 3: **The relationship between Marine Heatwaves and Archetype #4 in the Tasman Sea**: **a** snapshot of sea-surface temperatures (SST) anomalies for the peak of the 2016 strong marine heatwave event, which occurred on the 8th of February, 2016; and **b** SST composite average for all marine heatwave detected at a representative location. Statistics are calculated at the representative location 45.9°S,171°E, indicated by the grey circle. **c** the SST anomalies for best matching archetypal pattern (archetype 4); **d** time-series of SST anomalies (black) and the reconstruction from archetype 3 (orange) at the representative location shown in panels **a–c** ; **e** time-series of archetype affiliation probability for archetype 3. Colored bands in panels **d,e** indicate marine coldspell occurrences, coded by the severity category described in *Hobday et al. 2018* [3].

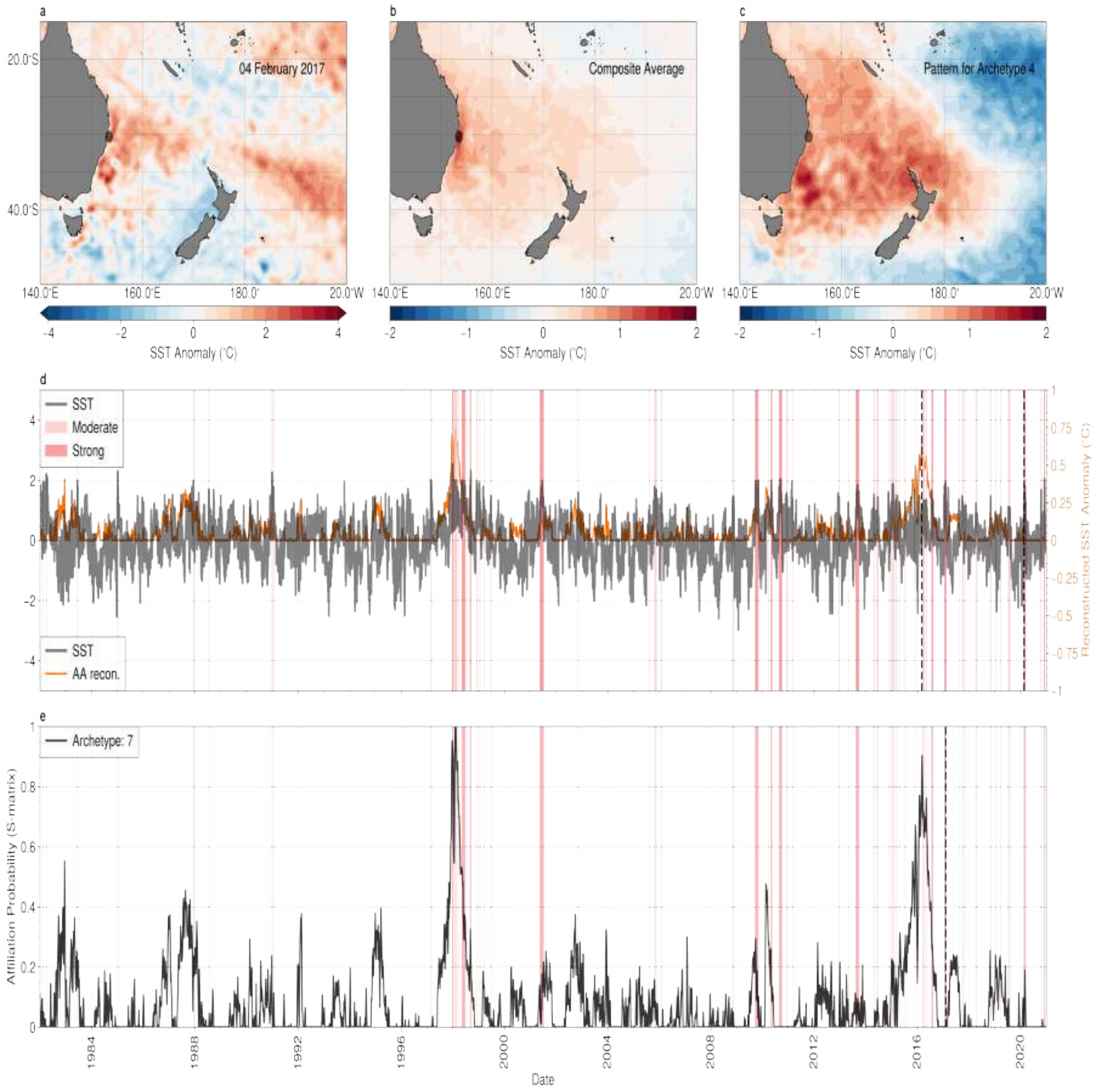

Supplementary Figure 4: **The relationship between Marine Heatwaves and Archetype #7 in the East Australian Current**: **a** snapshot of sea-surface temperature (SST) anomalies for the peak of the 2017-2018 severe marine heatwave event, which occurred on the 5th of December, 2017; and **b** SST composite average for all marine heatwave detected at a representative location. Statistics are calculated at the representative location 30°S,152°E, indicated by the grey circle. **c** the SST anomalies for best matching archetypal pattern (archetype 4); **d** time-series of SST anomalies (black) and the reconstruction from archetype 3 (orange) at the representative location shown in panels **a**–**c** ; **e** time-series of archetype affiliation probability for archetype 3. Colored bands in panels **d,e** indicate marine heatwaves occurrences, coded by the severity category described in *Hobday et al. 2018* [3]

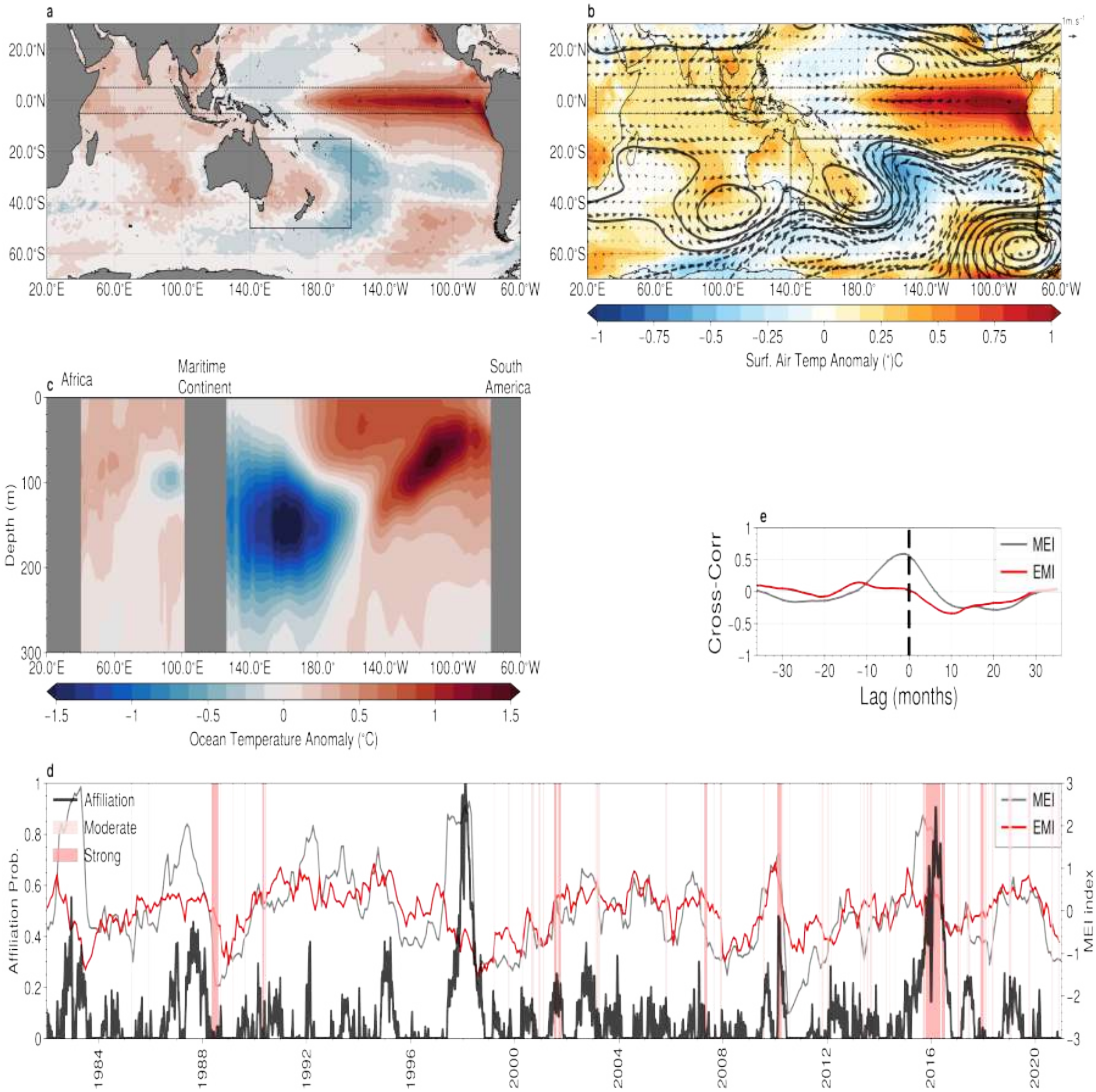

Supplementary Figure 5: **Teleconnections Associated with Tasman Sea Marine Heatwaves**: **a** Sea-surface temperature (SST) anomaly; **b** surface temperature (colours) with anomalous mid-tropospheric (500hPa) geopotential height (contour lines) and winds (vectors); and **c** equatorial subsurface temperatures, associated with archetype #6, the best-matching archetype for marine heatwaves in the southeast Indian Ocean.
textbfd The affiliation time-series (solid black) together with the multivariate El-Niño index (MEI, grey) and the Marshall Southern Annular Mode (SAM) index (blue). Periods of marine heatwaves are indicated by red shading. **e** the lagged cross-correlation between the affiliation time-series and the MEI (gray) and the Marshall Southern Annular Model (SAM) index (blue). Negative lags correspond to the MEI/SAM index leading the affiliation.

suggest that this archetype represents the variability associated with strong El-Niño events. Atmospheric circulation anomalies and sub-surface temperatures strongly support this inference, with the characteristic weaker than average trade winds evident in the atmospheric composites, and the warmer sub-surface ocean temperatures in the eastern Pacific and cooler than average temperatures in the western Pacific. However, the temperature expression is not particular strong in the Tasman Sea.

None of the 8 archetypes obtained by our analysis strongly projects into the Tasman Sea in the same way that other archetypes project into the south-east Indian ocean, and the archetype that projects the most strongly (Archetype #5) does not strongly reflect either the spatial or temporal structures of marine heatwaves in the region. Taken together, this leads us to conclude that in this complex western boundary current region, marine extremes are likely driven by local process not well captured by the large scale archetypes.

Supplementary Methods

Archetypal Patterns

In the main text, we show only 4 of a total of 8 archetypal patterns - a choice motivated by a desire to avoid clutter of both text and graphics. For completeness, the remaining archetypes are shown in Supplementary Fig. 6. The archetypes not shown in the main text are #5, #6, #7 and #8. Archetypes #1 and #2 (Supplementary Fig. 6**a,b** and Supplementary Fig. 6**c,d**) are discussed in the marine coldspell case study included within the this supplementary material. Here, archetypes labelling is arbitrary. However, archetypes can be ranked by a number of methods. For example, the most probable archetype can be computed by the summation of the affiliation matrix across all time steps, or the archetype that expresses highest variance can be computed by reconstructing the original data matrix (Eqn. 1 in the main text) archetype by archetype.

As in the main text, we also show the temporal occurrence and persistence of all 8 archetypes in Supplementary Fig. 7. The archetypes not discussed in the main text show periods of persistence, such as archetype #6 during the period 1993-1995, or archetype #8, which has the signature of moderate El-Niño and was strongly expressed in 1992 and 2007.

Time Shifted Composites of Archetypal Fields

As mentioned in the main text, the expression of a particular archetype may propagate temporally, and different regions may reach their maximum response to sometime before or after the a peak in the affiliation probability. Investigating the lagged response can provide insights into the temporal behaviour of the archetypal pattern.

Here, we plot the SST determined by calculating the weighted average of the SST and the affiliation time-series, shifted by -90, -60, -30, 0 and +15 days.

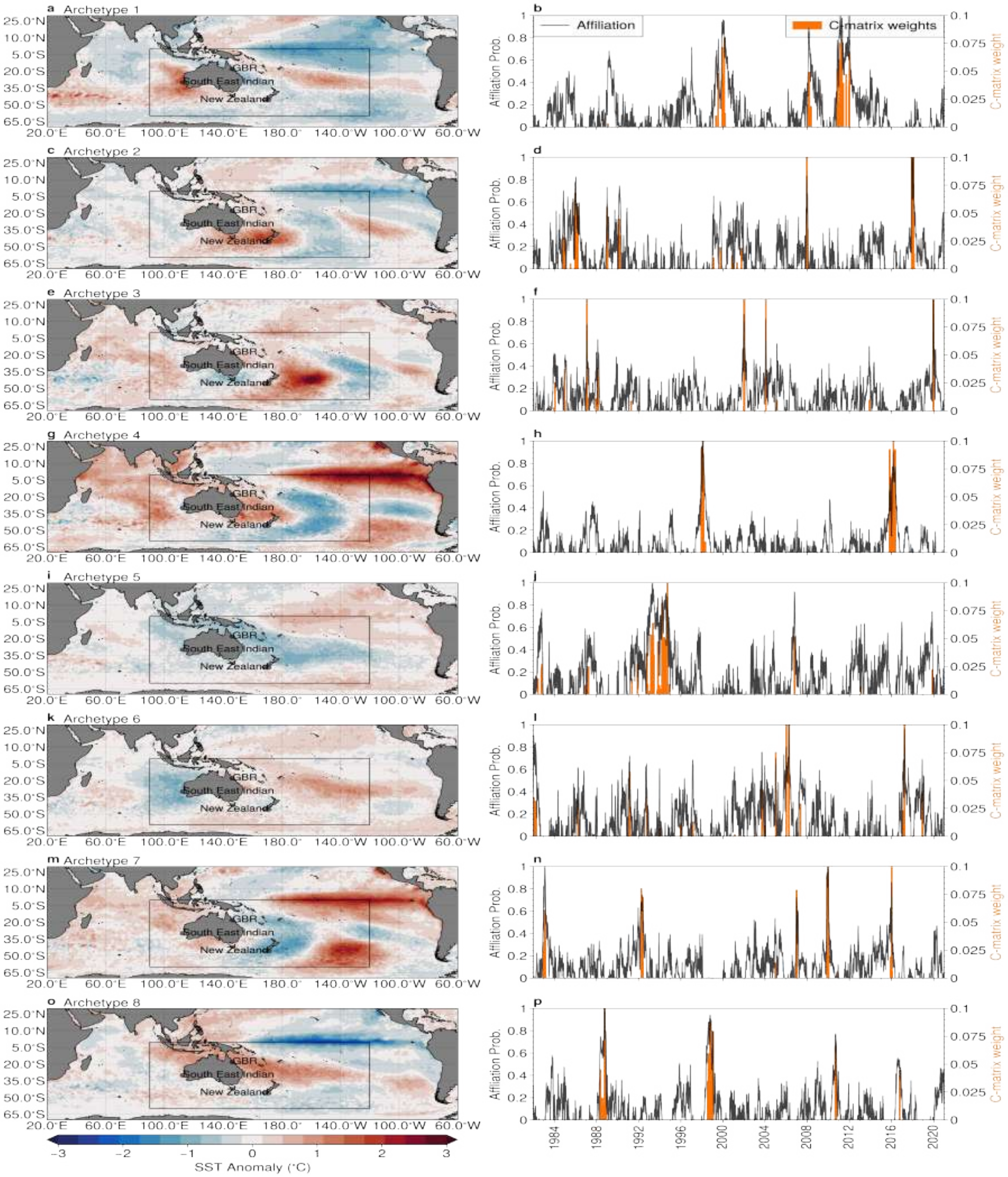

Supplementary Figure 6: **Archetypal Patterns and Affiliation time-series over the and South Pacific**: (left) Detrended sea-surface temperature (SST) anomalies for all eight archetypal patterns computed over the Australasian region (indicated by the black box), ranked from most likely to least likely to occur, and (right) associated affiliation time-series (black solid line) and the *C-matrix weights* applied to each time snapshot to form the archetypes (orange bars). The AA is conducted in the domain in the left-hand column. Archetypes #1 (panels **a,b**), #2 (panels **c,d**), #3 (panels **e,f**) and #4 (panels **a,b**) are used in the regional case studies in the main text (locations indicted in the text) while archetype #8 (panels **i,j**) is shown to illustrate classical El Niño type variability. Archetypes #5 (panels **i,j**), #6 (panels **k,l**), and #4 (panels **g,h**) are used in regional case studies in the supplementary material.

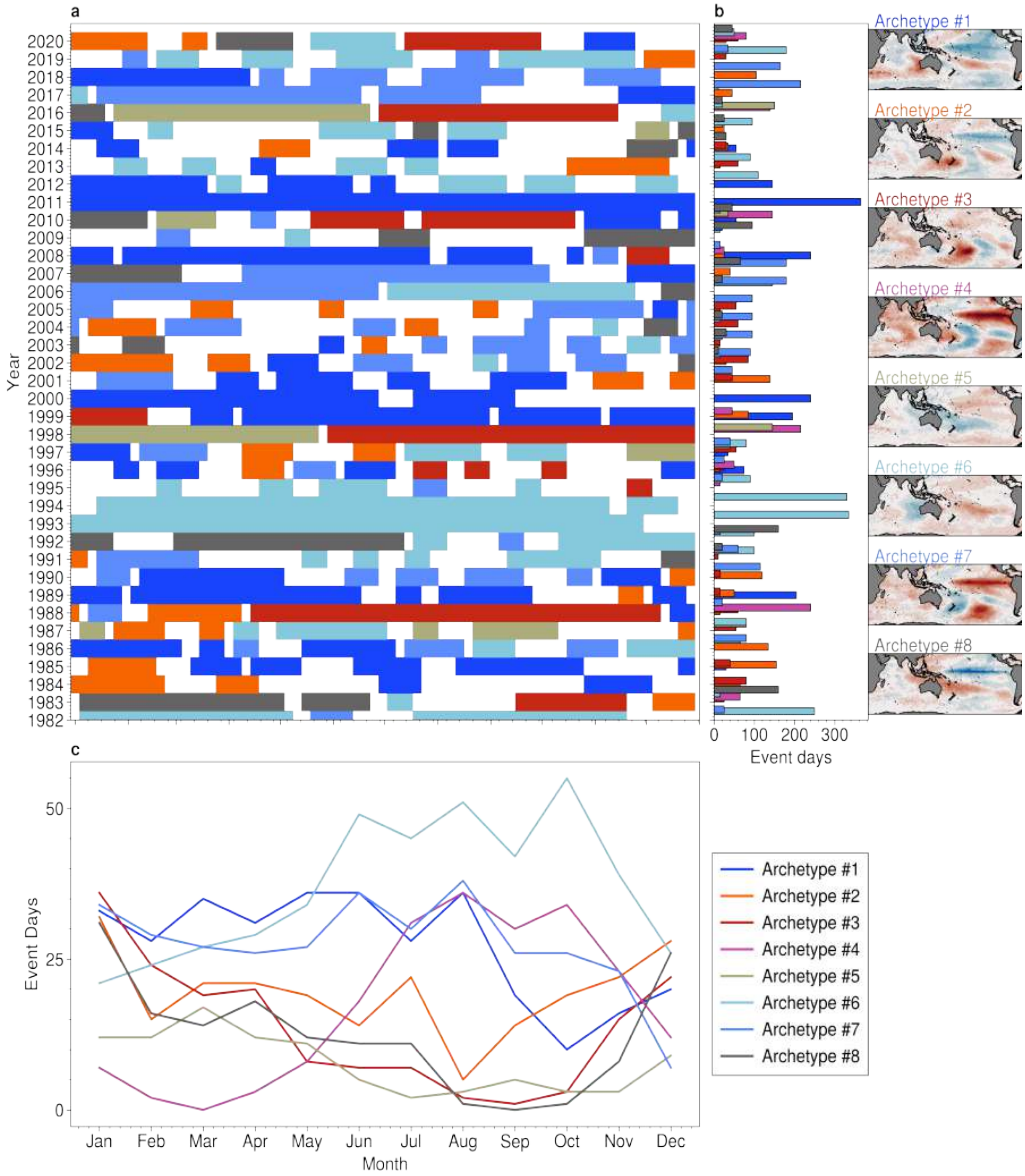

Supplementary Figure 7: **Temporal Occurrence of Archetype Patterns**: **a** Coloured blocks indicate period where a particular archetype was dominant for at least 20 days. The y-axis indicates the year, while the x-axis indicates the calendar days. Blanked periods show days where no qualifying event was found; the total number of archetype event days for each archetype that occur **b** for each year; and **c** for each 5-day period over the annual cycle. Maps to the right show spatial patterns of sea-surface temperature (SST) anomaly for each archetype.

We note strong persistence in certain archetypal patterns (for example, those associated with El-Niño or La-Niña like modes).

To better assess the temporal evolution of the extreme climate modes determined by archetype analysis, we plot the time-shifted composite SST for all archetypes at the representative location for each of the case-studies presented in the main text, in Supplementary Fig. 12. Together with the persistence plots shown in Supplementary Fig. 7, these results describe the time-scales of the evolution of each mode, as well as its phasing.

For the first case-study in the Southeast Indian Ocean (Supplementary Fig. 12**a**), it can be seen that the dominant (positive) influence on the SST in the region is archetype #1 (which corresponds to the *best-matching archetype* in the main text). It's influence is greatest at a lag of 0, and shows remarkable persistence, with a continued positive expression even 150 days prior to and after the peak of the event. In contrast, the New Zealand case-study (Supplementary Fig. 12**b**), shows that the best-matching archetype #2, has both a faster growth an decay rate, with an e-folding timescale of approximately 50 days. The differing timescales is consistent with the different dynamical origins of the marine heatwaves events: the slow evolution of tropical La-Niña conditions and their propagation through the Indonesian archipelago over the course of months in the southeast Indian case study, the (relatively) fast evolution of atmospheric blocking in the later.

In the final case study in the Great Barrier Reef region (Supplementary Fig. 12**c**) we note that archetype #4, associated with strong classical El-Niño like events, has its strongest influence on the representative location approximately 100 days *after* the strongest expression of the mode, centered in the equatorial Pacific. This suggests that the response of the GBR region to El-Niño occurs during the decay phase of the event. Archetype #3 shows only a weak response in the region leading the expression of the climate mode in the south Pacific by several days.

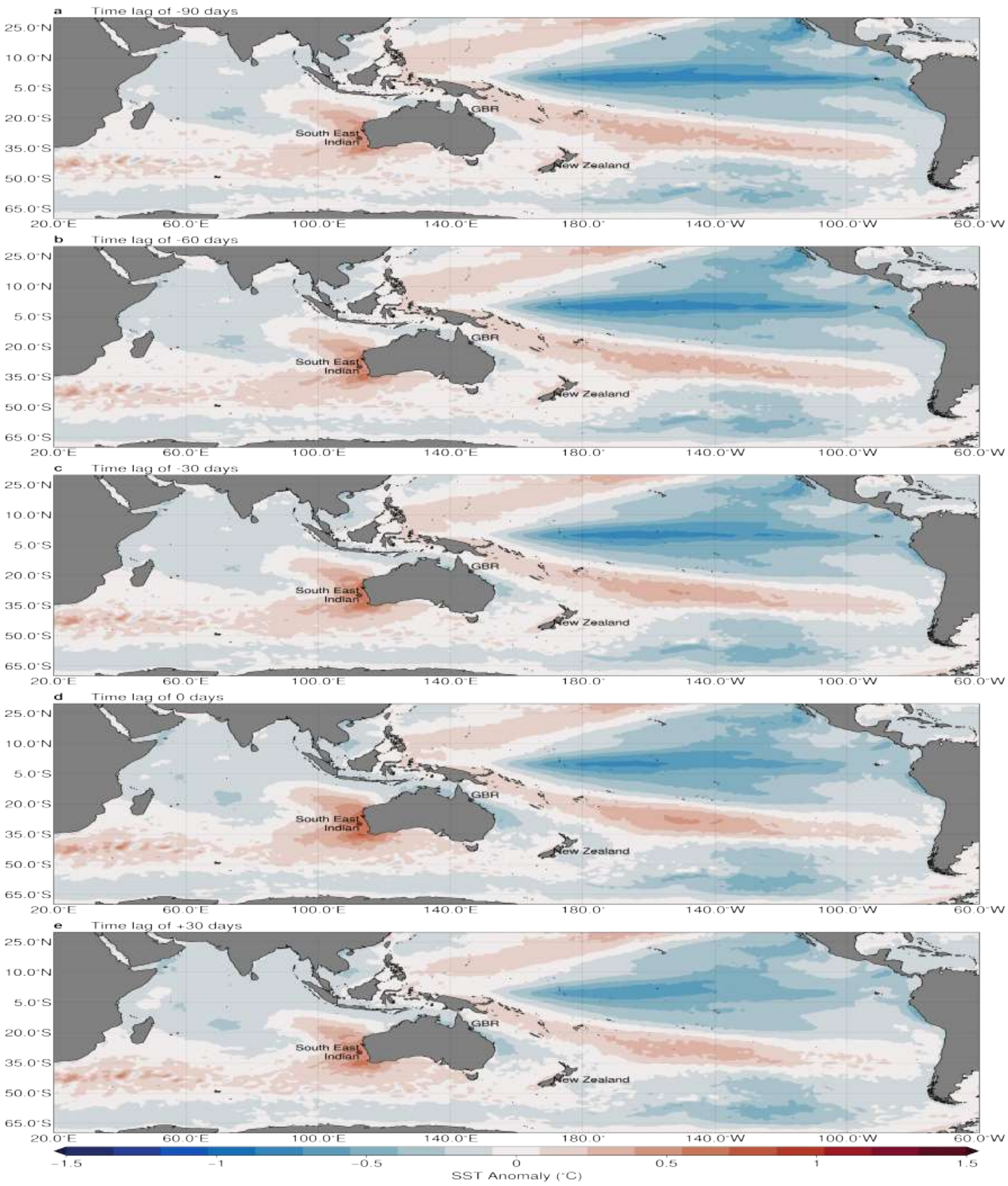

Supplementary Figure 8: **Sea-surface temperature lagged S-matrix composites for archetype #1**: **a** -90 days; **b** -30 days; **c** -30 days; **d** 0 days (identical to those plotted in the main text); and **e** +30 days.

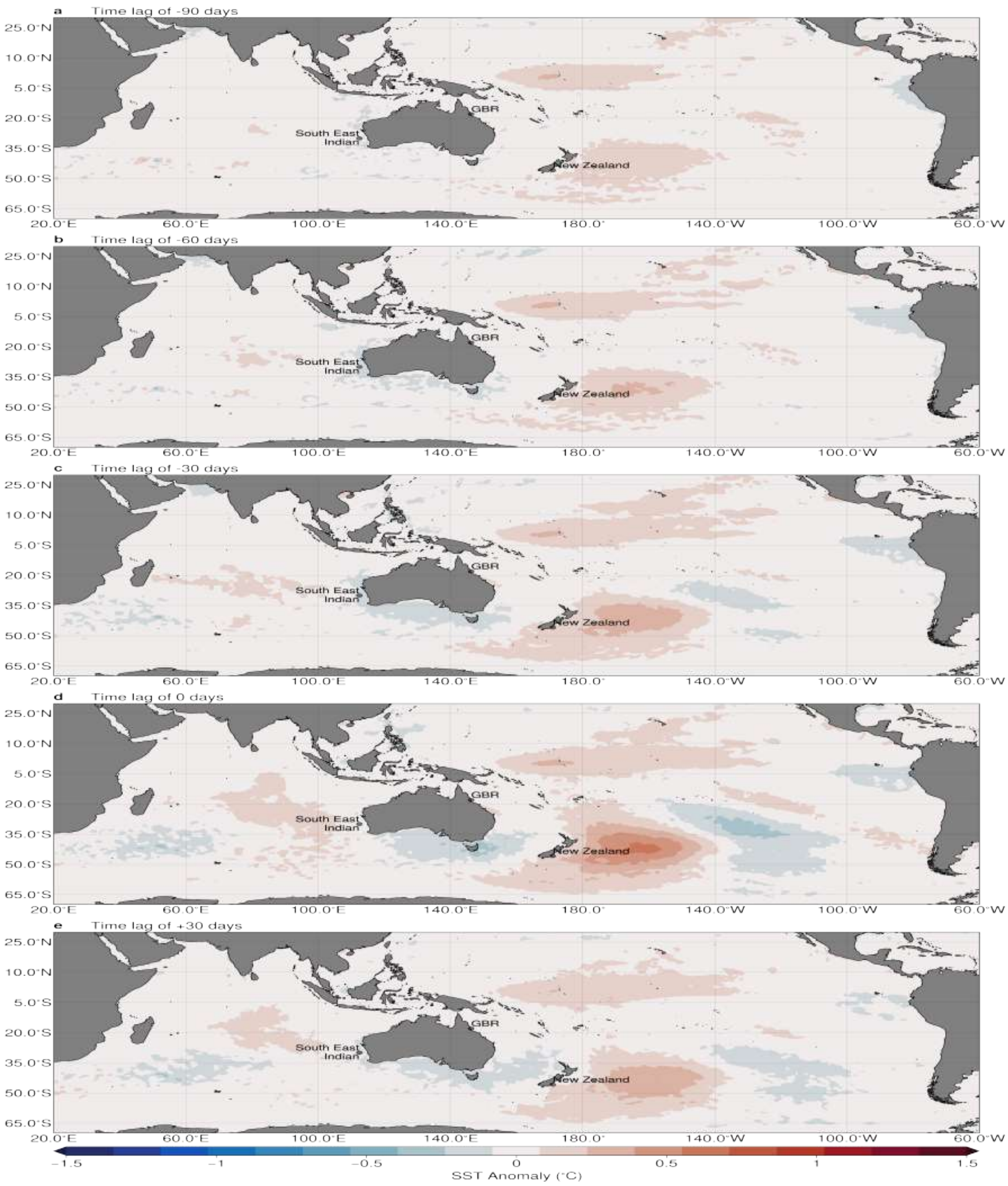

Supplementary Figure 9: **Sea-surface temperature lagged S-matrix composites for archetype #3**: **a** -90 days; **b** -30 days; **c** -30 days; **d** 0 days (identical to those plotted in the main text); and **e** +30 days.

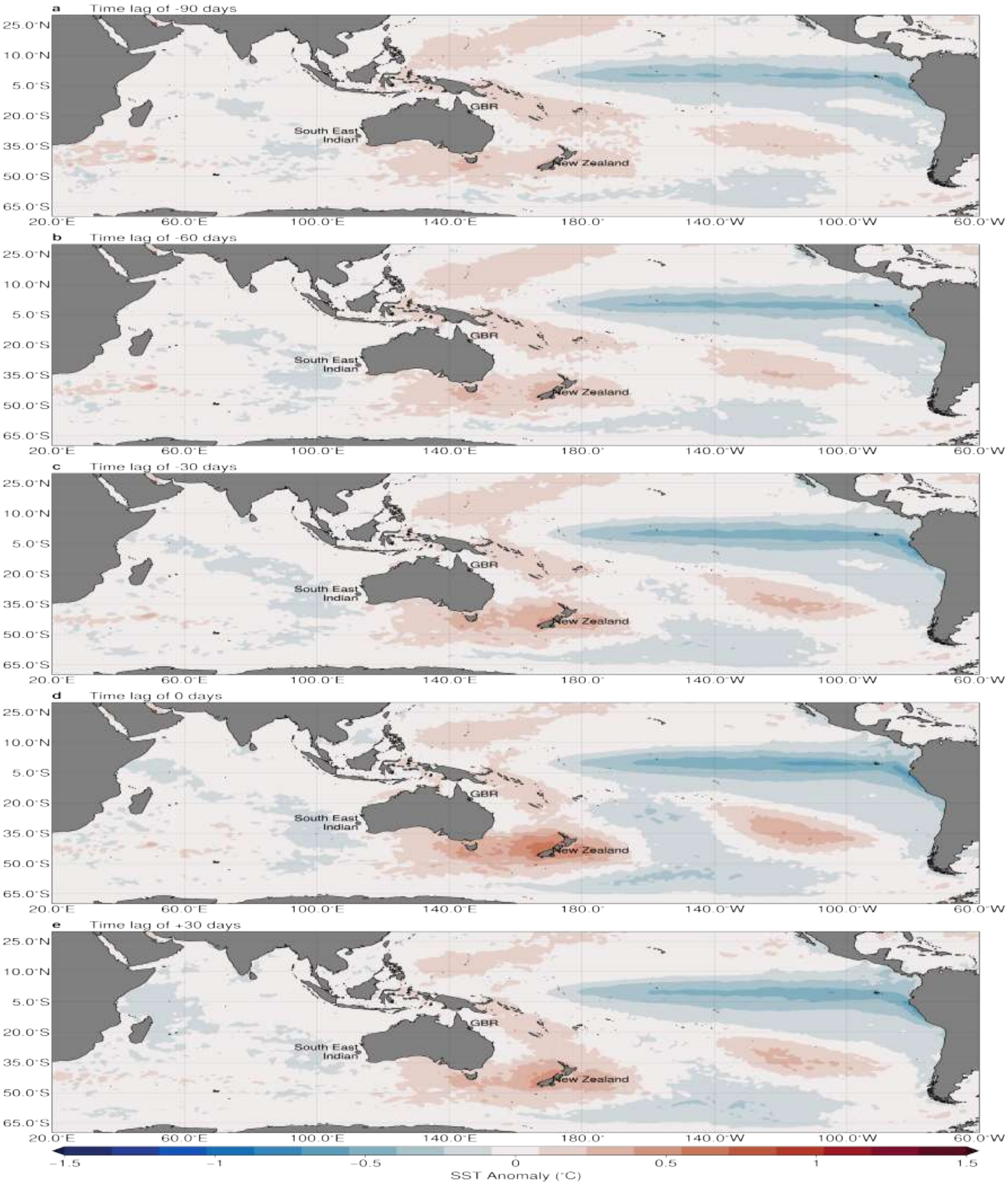

Supplementary Figure 10: **Sea-surface temperature lagged S-matrix composites for archetype #4**: **a** -90 days; **b** -30 days; **c** -30 days; **d** 0 days (identical to those plotted in the main text); and **e** +30 days.

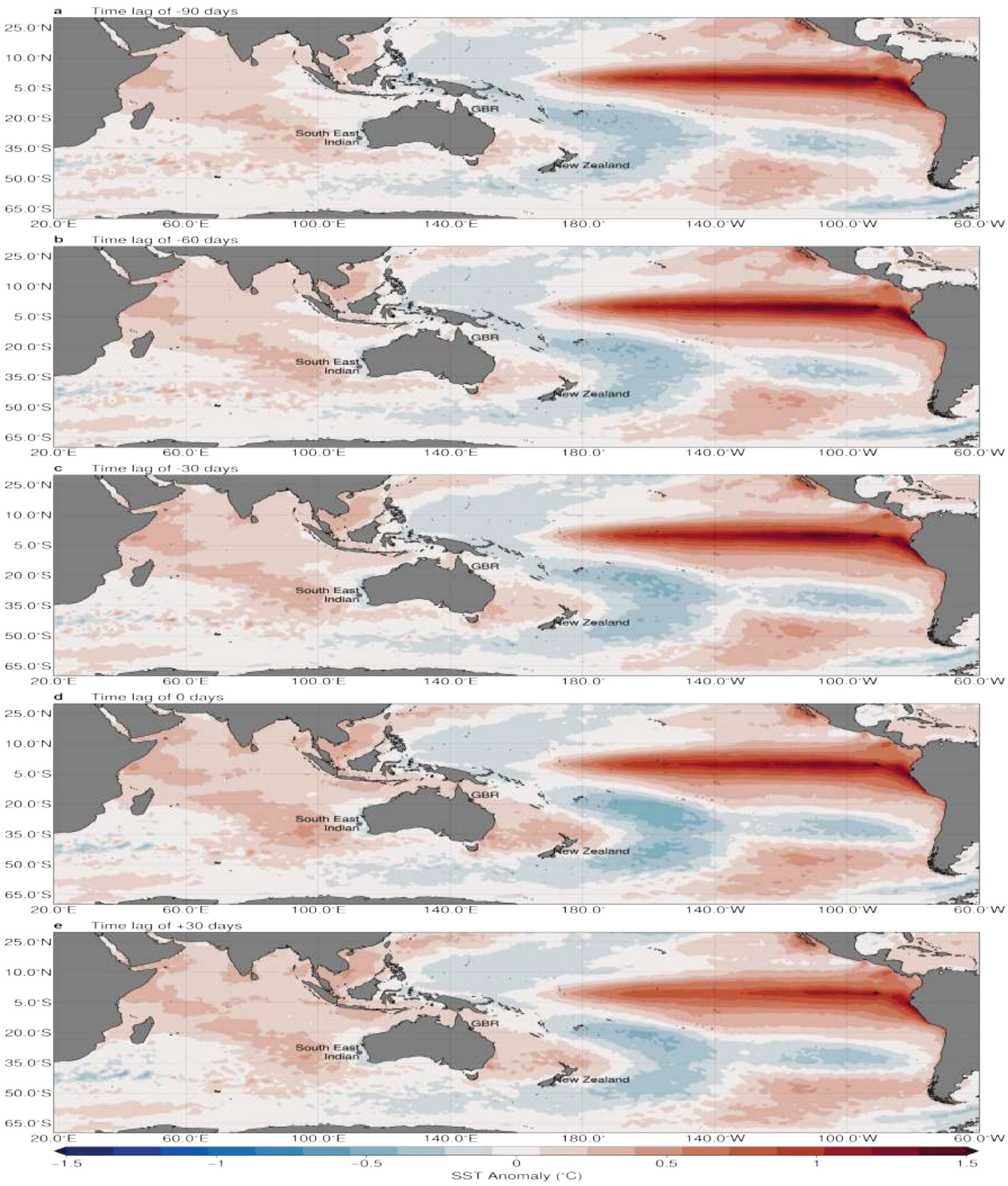

Supplementary Figure 11: **Sea-surface temperature lagged S-matrix composites for archetype #4**: **a** -90 days; **b** -30 days; **c** -30 days; **d** 0 days (identical to those plotted in the main text); and **e** +30 days.

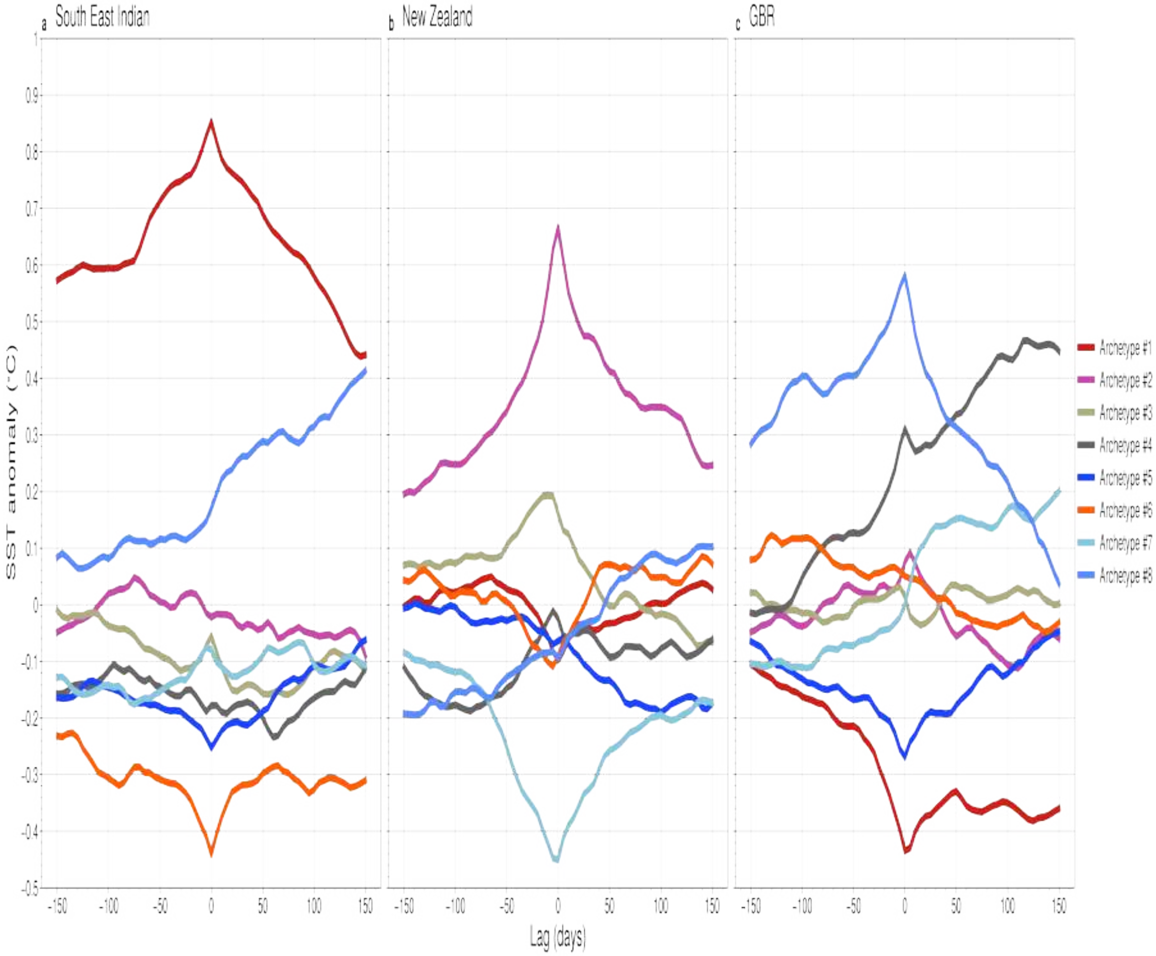

Supplementary Figure 12: **Lagged sea-surface temperature for each archetypal pattern at for each main text case study a** South-east Indian Ocean; **b** South-west Pacific/New Zealand; **c** Great Barrier Reef region.

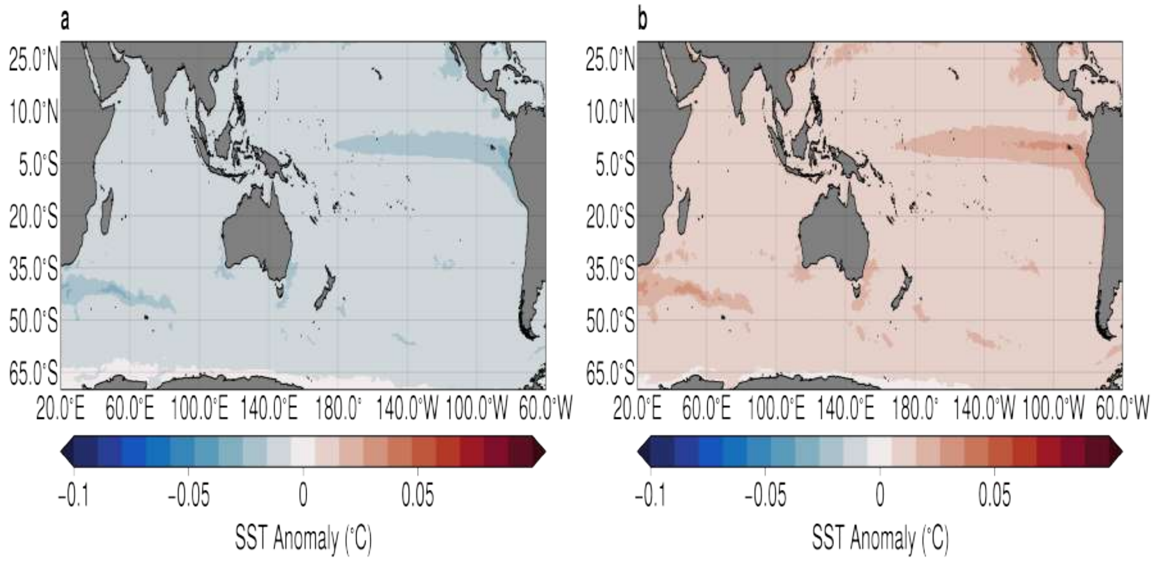

Supplementary Figure 13: **Quantiles of sea-surface temperature used for statistical significance testing**: **a** 5th percentile; and **b** 95th percentile.

Statistical Significance of Archetypal Patterns

In order to evaluate the statistical significance of archetypal spatial patterns and the composite fields, we employ a brute-force Monte-Carlo approach. First, we generate synthetic stochastic matrix, designed to replicate the features of the C or S matrices. The elements of these matrices is drawn from a uniform distribution between 0 and 1. The rows or columns of these synthetic stochastic matrices are them normalised appropriately to ensure that the constraints. For example, in the case of a synthetic C-matrix, the normalisation is applied to rows to ensure that the constraint $\sum_t^T c_{t,j} = 1$ is satisfied. In the case of a synthetic S-matrix, the normalisation is performed column-wise, to satisfy the constraint $\sum_j^P s_{t,j} = 1$. We then form composite fields on these synthetic matrices. The procedure is repeated 1000 times and the 5th and 95th percentiles computed (shown in Supplementary Fig. 13). A pixel is declared 'significant' if it is less than the 5th percentile, or greater than the 95th percentile.

In Supplementary Fig. 14, we plot the SST archetypal patterns for all 8 archetypes, similarly to Supplementary Fig. 6. Stippling indicates statistical significance. As can be seen in the figure, fields are almost everywhere statistically significant (with the exception of regions that separate warm and cold anomalies). This result might be expected from the fact that AA identifies extreme states.

In Supplementary Fig. 15, we show the statistical significance of the geopo-

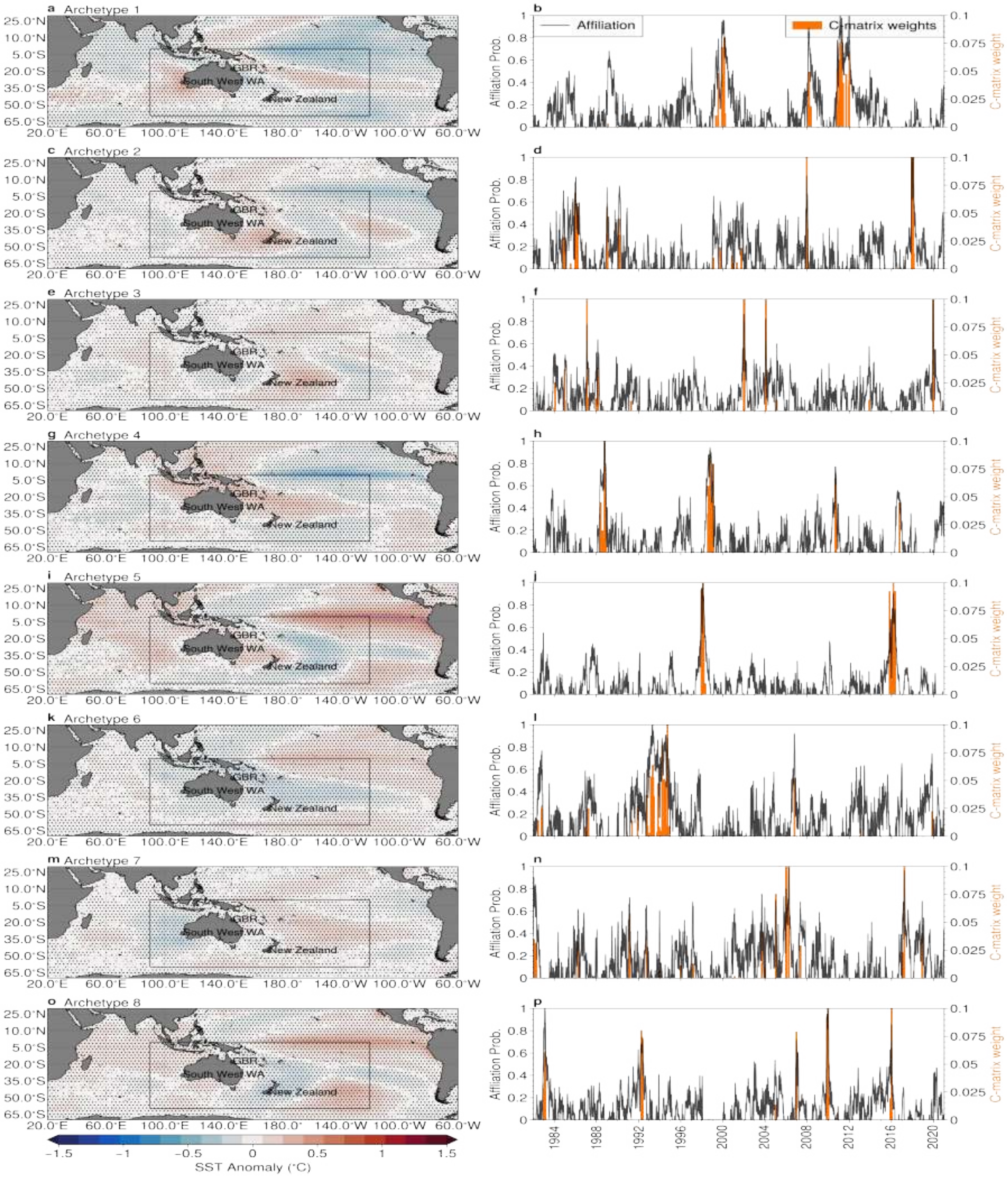

Supplementary Figure 14: **Statistical Significance of Archetypal sea-surface temperature Patterns** : (left) Detrended sea-surface temperature (SST) anomalies for all eight archetypal patterns computed over the Australasian region (indicated by the black box), and (right) associated affiliation time-series (black solid line) and the *C-matrix weights* applied to each time snapshot to form the archetypes (orange bars). Stippling indicates regions where the patterns are statistically significant at the 95% confidence level (right) associated affiliation time-series (black solid line) and the *C-matrix weights* applied to each time snapshot to form the archetypes (orange bars)

tential height patterns computed using the S-matrix weights obtained from the archetypes computed using SST. Unlike the SST patterns themselves, the geopotential height patterns are not everywhere significant. However, the regions of statistical significance typically encapsulate the broad-scale teleconnections and larger flow features.

To conclude, the spatial patterns produced by the AA undertaken in this study are robust and, nearly everywhere, statistically significant. We have also tested the statistical significance of surface atmosphere temperature and subsurface oceanic temperatures. However, these are not shown for brevity. The conclusions however, are the same, all major features of the identified archetypal or composite fields are significant at the 95% confidence level. However, it is worth mentioning that there is, to the best of our knowledge, no consensus on the best statistical significance test for archetype analysis. In general, rather than sampling points randomly from the distribution, one should sample points close to the convex hull of the dataset, and test those points against the "corner" points determined by AA. However, specification of the convex hull of a high dimensional dataset is difficult, both conceptually and computationally. As such, in this study we have opted to use a conceptually simple approach to significance testing, noting that it may not be optimal for AA.

Supplementary References

[1] Alan F. Pearce and Ming Feng. The rise and fall of the "marine heat wave" off western australia during the summer of 2010/2011. *Journal of Marine Systems*, 111-112:139–156, 2013.

[2] Ming Feng, Nick Caputi, Arani Chandrapavan, Miaoju Chen, Anthony Hart, and Mervi Kangas. Multi–year marine cold-spells off the west coast of australia and effects on fisheries. *Journal of Marine Systems*, 214:103473, 2021.

[3] Alistair J. Hobday, Eric C.J. Oliver, Alex Sen Gupta, Jessica A. Benthuysen, Michael T. Burrows, Markus G. Donat, Neil J. Holbrook, Pippa J. Moore, Mads S. Thomsen, Thomas Wernberg, and Dan A. Smale. Categorizing and naming marine heatwaves. *Oceanography*, 31, June 2018.

[4] Eric Oliver, Jessica Benthuysen, Nathaniel Bindoff, Alistair Hobday, Neil Holbrook, Craig Mundy, and Sarah Perkins-Kirkpatrick. The unprecedented 2015/16 tasman sea marine heatwave. *Nature Communications*, 8:16101, 07 2017.

[5] A. Schaeffer and M. Roughan. Subsurface intensification of marine heatwaves off southeastern australia: The role of stratification and local winds. *Geophysical Research Letters*, 44(10):5025–5033, 2017.

[6] Alistair Hobday and Gretta Pecl. Identification of global marine hotspots: Sentinels for change and vanguards for adaptation action. *Reviews in Fish Biology and Fisheries*, 24, 06 2014.

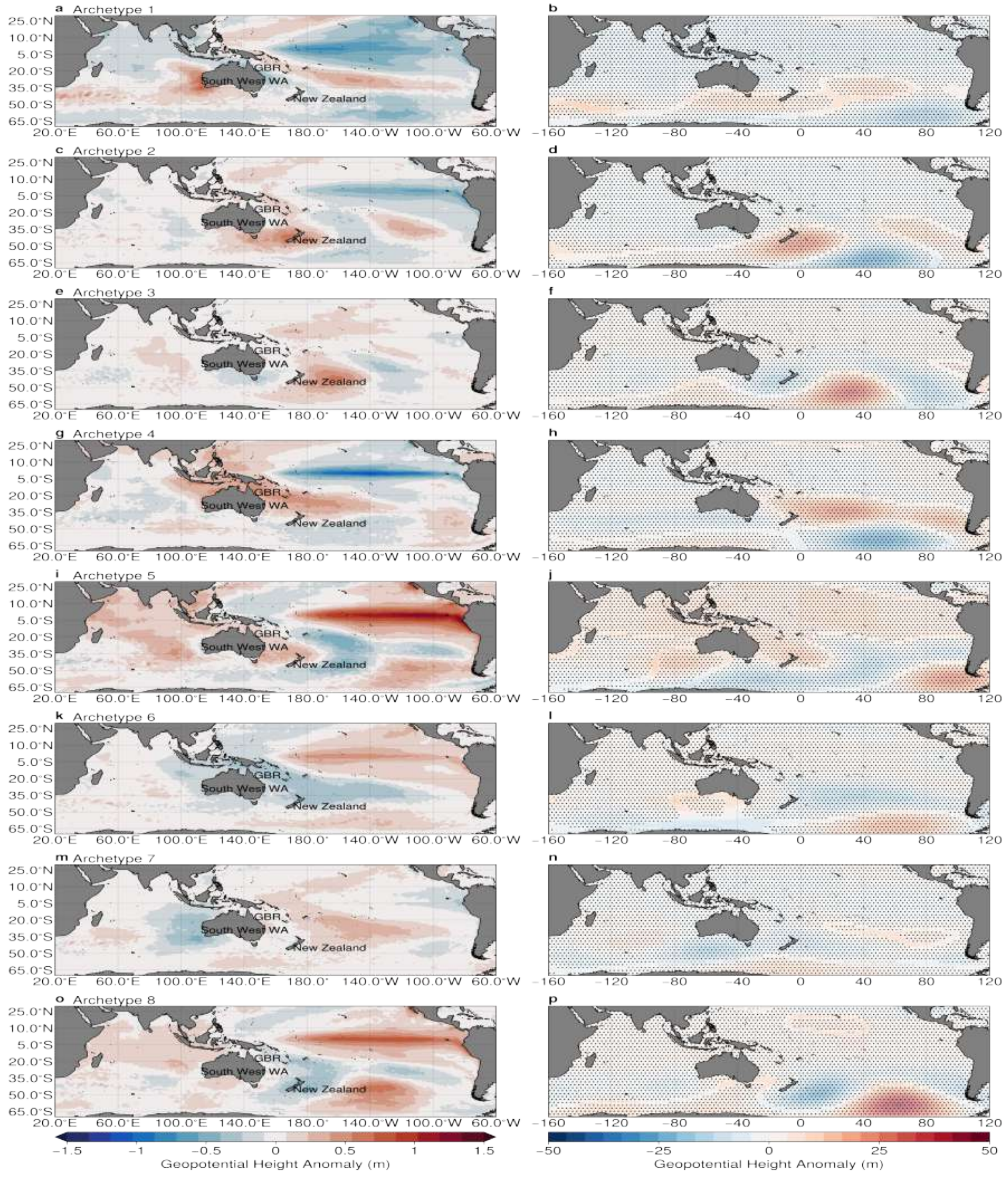

Supplementary Figure 15: **Statistical Significance of Archetypal 500hPa Geopotential Patterns** : (left) Detrended sea-surface temperature (SST) anomalies for all eight archetypal patterns computed over the Australasian region (indicated by the black box), and (right) associated mid-tropospheric 500hPa geopotential height anomalies. Stippling indicates regions where the patterns are statistically significant at the 95% confidence level
